# The Genome of a Pigeonpea Compatible Rhizobial Strain ‘10ap3’ Appears to Lack Common Nodulation Genes

**DOI:** 10.3390/genes14051084

**Published:** 2023-05-14

**Authors:** Francina L. Bopape, Ahmed Idris Hassen, Rogerio M. Chiulele, Addmore Shonhai, Eastonce T. Gwata

**Affiliations:** 1 Agricultural Research Council, Plant Health and Protection (ARC-PHP), Private Bag X134, Pretoria 0121, South Africa; phalanef@arc.agric.za (F.L.B.); hassena@arc.agric.za (A.I.H.); 2 Department of Plant and Soil Sciences, Faculty of Science, Engineering and Agriculture, University of Venda, Private Bag X5050, Thohoyandou 0950, South Africa; 3 Centre of Excellence in Agri-Food Systems and Nutrition, Eduardo Mondlane University, 5th Floor, Rectory Building, 25th June Square, Maputo 1100, Mozambique; chiulele.rogerio@gmail.com; 4 Faculty of Agronomy and Forestry Engineering, Eduardo Mondlane University, Julius Nyerere Avenue, Maputo 1100, Mozambique; 5 Department of Biochemistry and Microbiology, Faculty of Science, Engineering and Agriculture, University of Venda, Private Bag X5050, Thohoyandou 0950, South Africa; addmore.shonhai@univen.ac.za

**Keywords:** circular chromosome, genome, nitrogen fixation, purine derivative, symbiosis

## Abstract

The symbiotic fixation of atmospheric nitrogen (N) in root nodules of tropical legumes such as pigeonpea (*Cajanus cajan*) is a complex process, which is regulated by multiple genetic factors at the host plant genotype microsymbiont interface. The process involves multiple genes with various modes of action and is accomplished only when both organisms are compatible. Therefore, it is necessary to develop tools for the genetic manipulation of the host or bacterium towards improving N fixation. In this study, we sequenced the genome of a robust rhizobial strain, *Rhizobium tropici* ‘10ap3’ that was compatible with pigeonpea, and we determined its genome size. The genome consisted of a large circular chromosome (6,297,373 bp) and contained 6013 genes of which 99.13% were coding sequences. However only 5833 of the genes were associated with proteins that could be assigned to specific functions. The genes for nitrogen, phosphorus and iron metabolism, stress response and the adenosine monophosphate nucleoside for purine conversion were present in the genome. However, the genome contained no common *nod* genes, suggesting that an alternative pathway involving a purine derivative was involved in the symbiotic association with pigeonpea.

## 1. Introduction

Pigeonpea (*Cajanus cajan* (L.) Millsp.) is an important grain legume which is cultivated mainly for human consumption as a valuable source of vitamins, minerals and protein [1,2,3]. It is highly tolerant to drought and suitable for production in semi-arid regions in Africa [4,5]. The pigeonpea plant also improves soil fertility through symbiotic nitrogen (N) fixation by associating with a diverse range of soil rhizobia [6]. The N is critical for plant growth and grain yield production.

Pigeonpea is generally compatible with soil rhizobia that belong to the Cowpea Miscellany Group, which commonly nodulate other legumes such as cowpea (*Vigna unguiculata*) and peanut (*Arachis hypogaea*) [7,8]. However, despite the promiscuous association with indigenous soil bacteria, there is variation in the symbiotic efficiencies among rhizobial strains that are compatible with pigeonpea. For instance, different strains from the genus *Bradyrhizobium* exhibited significant variation in N fixation efficiency in several legumes including the bambara groundnut (*Vigna subterranea*) and pigeonpea [7,9]. However, the reason for such variations among rhizobial strains remains unclear. Moreover, the symbiotic N fixation process involves multiple genes with various modes of action and is accomplished only when both organisms are compatible. The initial step requires a mutual exchange of molecular signals prior to the formation of both the root nodule and symbiosome, within which the rhizobia differentiate further into N fixing bacteroids [10,11]. Three additional genes of the rhizobia, namely, *nod*, *nif* and *fix*, control the fixation process. The *nod* and *nif* proteins are encoded by accessory bacterial genes that are housed in genetic elements (for instance, plasmids and chromids) that can be transmitted from one generation to the other. All the three genes are transferable horizontally in high frequencies within the bacterial species and infrequently between genera [12]. The *nif*A gene functions as a transcription regulator of other *nif* genes. The rhizobial signal molecules (or *Nod* factors), also called lipo-chito-oligosaccharides (LCOs), are encoded by a unique set of rhizobial genes called *nod* genes which induce host responses resulting in nodule formation [13]. On the other hand, the host plants secrete a variety of substances, which include flavonoids and phenolic compounds into the rhizosphere, in conjunction with the bacterial activator protein *Nod*D followed by the expression of rhizobial nodulation genes *nod*, *nol* and *noe* [14,15]. Because of the phylogenetic diversity of both the rhizobia and the host legume, there is no documented evidence of rhizobial strains that can form symbiotic relationships with all the legumes and vice versa. In most cases, there is specificity, which occurs at both the species and genotypic levels at different stages of the fixation process [10,13,16]. In addition, incompatibility can occur at a late stage of nodule development, resulting in diminished efficiency in N fixation [17,18]. Therefore, it is necessary to develop tools for the genetic manipulation of the host or bacteria to improve N fixation efficiencies. In this regard, knowledge of the genetic and molecular basis of the genetic material is essential. Specifically, the genome sizes of the different rhizobial species may provide some additional insights into the variation in symbiotic efficiencies of host plant genotype x rhizobial strain combinations.

Currently, the whole genome sequencing (WGS) method is a routine tool for genomics research which can generate large volumes of data that require powerful algorithms to accurately determine intrinsic genetic information such as the structural or copy number variations as well as breakpoints and sizes of genes [19]. Such information is useful in understanding the functional properties of rhizobial genes. Therefore, the objectives of this study were to (i) sequence the genome of a selected rhizobial strain derived from pigeonpea, (ii) determine the genome size of the rhizobial strain and (iii) identify some of the key genes in the genome of the strain.

## 2. Materials and Methods

### 2.1. Rhizobial Strain and DNA Extraction

The rhizobial strain *Rhizobium* sp. ‘10ap3’, originating from pigeonpea, was used in this study [6]. The strain was trapped previously from a soil sample that was collected from a location in South Africa and subsequently showed superior symbiotic efficiency with pigeonpea as measured by the nodule and shoot dry weight [6,20]. The *Rhizobium* sp. ‘10ap3’ was identified by the sequencing of its 16S rRNA and *rec*A gene [6]. The strain was revived by streaking on yeast mannitol agar (YMA) with Congo red and incubated at 28 °C for 3 days. For DNA extraction, a single pure colony of the bacterium grown on YMA was transferred to tryptone yeast broth medium and incubated for 3 days on a rotary shaker (150 rpm) at 28 °C. A total of 1.5 mL of the broth culture was used to extract DNA using the Wizard genomic DNA extraction kit per the manufacturer’s instructions (Promega, Madison, WI, USA) for use in NGS. DNA concentration to 0.2 ng/μL was quantified by adjusting and diluting with a required volume of distilled water. For Nextera XT tagment amplicon construction, 5 μL tagmentation DNA buffer and 2.5 μL amplification tagmentation were mixed with 2.5 μL input DNA (0.2 ng/μL) in a PCR tube. The samples were transferred to a thermocycler, programmed for one-step at 55 °C for 5 min, with heated lid, followed by hold at 10 °C for a 10 μL volume. This was followed by neutralizing NTA by adding 2.5 μL neutralization tagmentation buffer and incubation for 5 min at room temperature [21]. The *Rhizobium* sp. ‘10ap3’ was identified previously by sequencing its 16S rRNA and *recA* gene [6].

### 2.2. Sequencing, DNA Libraries and Cluster Generation

PCR amplification and clean-up of the PCR product method was similar to the protocol that was used previously [21]. Sequencing was carried out as per the MiSeq sequencing guide by Illumina for the MiSeqTM System platform (Illumina Corporation, San Diego, CA, USA) with a 2 × 300 bp double-ended sequencing strategy using the MiSeq Reagent Kit v3. The MiSeq System consists of on-board data analysis and access to the Illumina genomic cloud-computing platform BaseSpace™ Sequence Hub. We used the DNA Library Prep Kit for Illumina (CD NEXT), which can integrate complex steps of DNA fragmentation, end repair and dA-tailing as well as adapter ligation into a one-step enzymatic reaction, with the capacity to utilize library preparation from as low as 1 ng of starting material. DNA libraries were prepared using the Nextera protocol (Illumina, San Diego, CA, USA) and paired-end (300 bp × 2) sequenced on a MiSeq (Illumina) sequencer at the Biotechnology Platform, Agricultural Research Council–Onderstepoort Campus, Pretoria, South Africa. The sample preparation process included the addition of adaptors to the end of the DNA fragments. The adapter sequences and low-quality bases were trimmed, and the overlapping pairs were merged using CLC Genomic Workbench version 8.5.1, resulting in 8,265,062 reads that were used in a de novo assembly that produced the draft genome sequence. The gene annotations were performed with the NCBI Prokaryotic Genome Annotation Pipeline [22]. Sequencing binding sites, such as indices and regions complementary to the flow cell oligonucleotides, were introduced to the fragments, allowing the DNA to bind to the flow cell prior to amplification and purification [23].

The libraries or samples are loaded on to the flow cell and placed on the sequencer. The clusters of DNA fragments are amplified by cluster generation that results in millions of copies of single stranded DNA. Cluster generation is a feature that is associated with most Illumina sequencing instruments and initiates with hybridization, which is enabled by any of the two oligonucleotides on the surfaces of the flow cell. The polymerase then creates a complement of hybridized fragment. Pooled libraries were prepared for loading on the MiSeq (Illumina) sequencer and the bioinformatics analyses were conducted using software (CLC Genomic Workbench version 8.5.1) for the analysis of the WGS data [22,23,24].

## 3. Results

### 3.1. Genome Sequencing and Size

**Figure 1:**
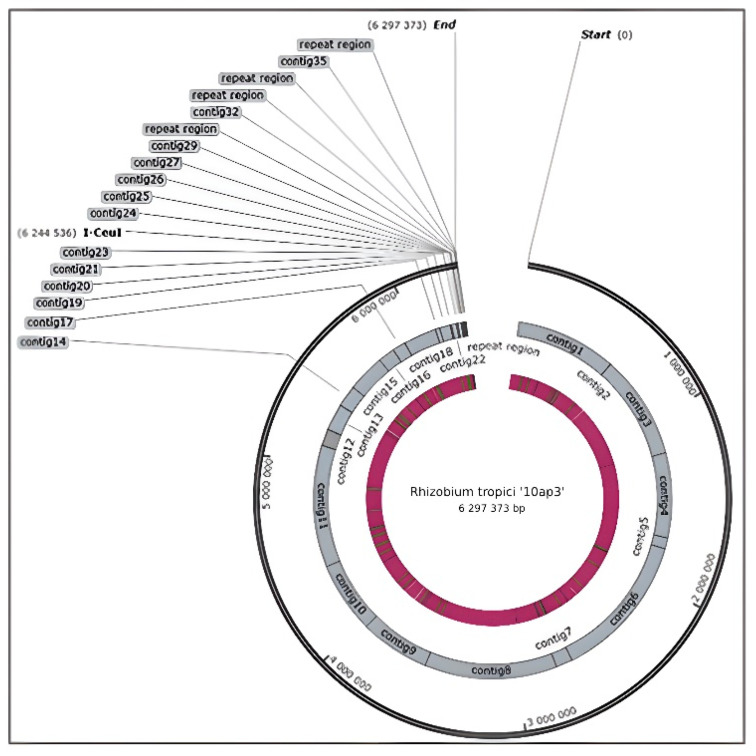
Snap gene circular view of a 6.3 MB genome of strain ‘10ap3’ showing all the contigs that resulted from the RAST annotation of the genome assembly.

The sequencing of the ‘10ap3’ strain genome resulted in 10,364,436 raw sequence reads, which further resulted in 8,265,062 reads when trimming and adapter adding was complete. Upon successful sequencing of the whole genome of the strain, it was allocated the accession number VNIP00000000 and deposited into the DNA DataBank of the Japan/European Nucleotide Archive/Genbank at NCBI for further reference and use (https://www.ncbi.nlm.nih.gov/search/all/?term=VNIP00000000 (accessed on 30 September, 2022). The genome was a large chromosome with a circular shape (Figure 1) and an overall G + C content of 60.0%. The size of the genome of the strain was 6,297,373 bp and consisted of 36 contigs (including scaffolds) that were obtained after assembly. The maximum scaffold size was 876,087 bp, consisting of several contigs each coding a protein. Furthermore, additional features were identified on the genome, including 45 tRNAs (Table 1).

### 3.2. Key Genes in the Genome

**Figure 2:**
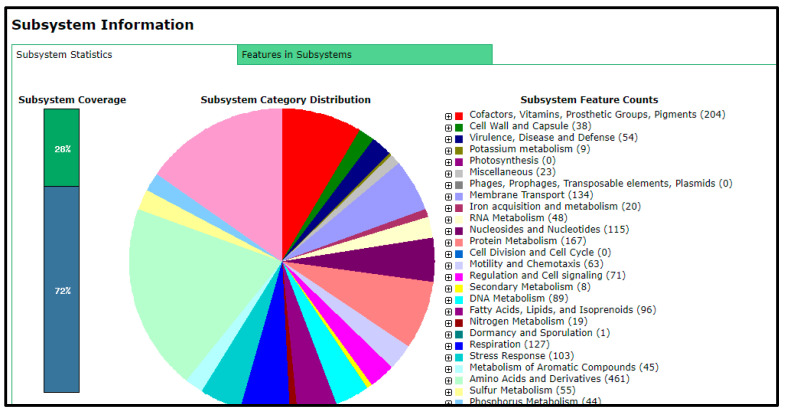
Graphical representation of the functions coded on the whole genome of the rhizobial strain ‘10ap3’.

**Figure 3:**
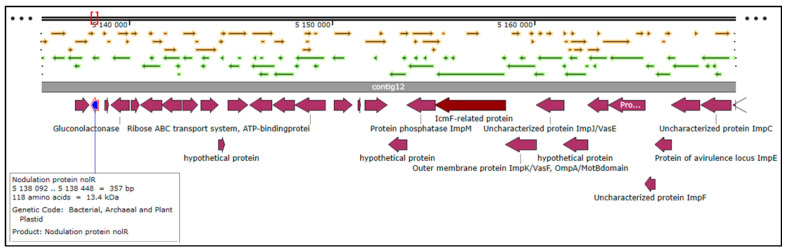
Sections of the chromosomal region of the rhizobial strain ‘10ap3’ showing the open reading frames, contigs, coding regions and the nodulation protein (*nolR*) located on contig 12. Different arrows on the figure represent genes on the chromosome and the selected genes of interest are highlighted with different colors on the CDs just below the contig 12. Different arrows on the figure represent genes on the chromosome and the selected genes of interest are highlighted with different colors below the contigs.

**Figure 4:**
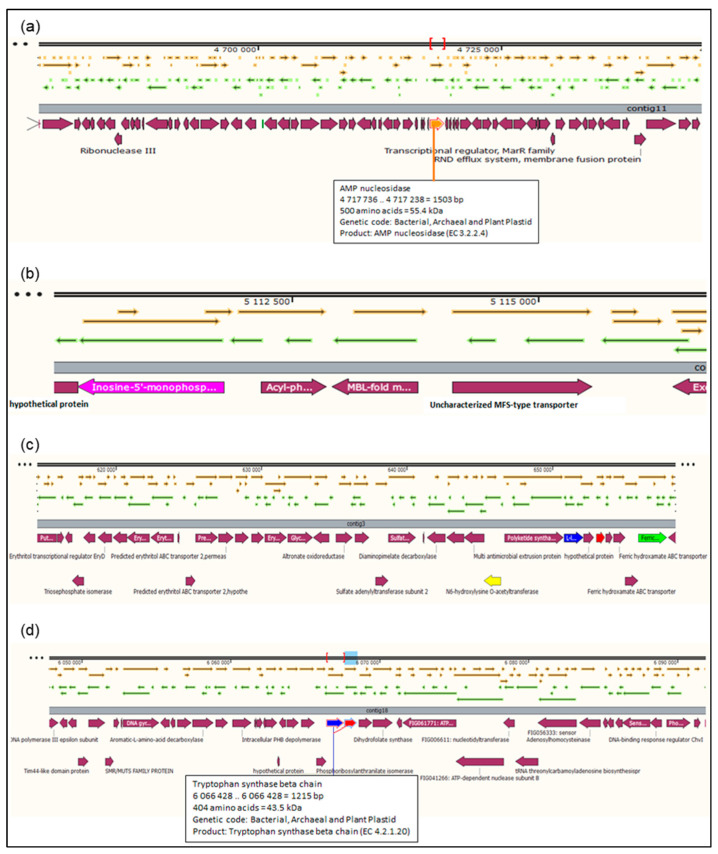
Sections of the chromosomal region of the rhizobial strain ‘10ap3’ showing open reading frames, contigs and coding regions (CDs). Different arrows on the figure represent genes on the chromosome and the selected genes of interest are highlighted with different colors on the CDs just below the contigs: (**a**) adenosine monophosphate (AMP) nuclease, purine conversion located on contig 11, (**b**) inosine-5-monophosphate located on contig 11, (**c**) siderophores aerobactin synthesis genes located on contig 3 and (**d**) auxin biosynthesis genes, tryptophan synthase α and β chains located on contig 18. Different arrows on the figure represent genes on the chromosome and the selected genes of interest are highlighted with different colors below the contigs.

The total number of genes in the rhizobial strain ‘10ap3’ genome were 6013, of which 99.13% were coding sequences (or protein-coding genes). However, only 5833 of the protein-coding genes had proteins that could be assigned to specific functions. Several important genes that were found in the genome included those involved in nitrogen metabolism, stress response, phosphorus metabolism and iron acquisition as well as some additional ones such as siderophore aerobactin synthesis genes, inosine-5-monophosphate for purine conversion, adenosine monophosphate nucleoside for purine conversion and auxin biosynthesis genes for tryptophan synthase α and β chains (Figure 2). The nodulation gene *nolR*, which functions as a DNA binding transcription factor, was present in the genome (Figure 3). Precursor genes for purine synthesis inosine-5-monophosphate and adenylosuccinate, which are responsible for nodule formation, were also present in the genome (Figure 4).

## 4. Discussion

The results of this study demonstrated the successful sequencing of the genome of a rhizobial strain that was originally isolated from pigeonpea root nodules. The genome of the rhizobial strain ‘10ap3’ does not contain common *nod* and *ni*f genes. In comparison with similar studies, the genomes of other strains of *R. tropici* such as strain CIAT899 contains most of the nod and *nif* genes while those of the other five strains shared only two common genes, namely the nod *nfe*D for amino the deoxy-chrorismate synthatase component and the *nif*U gene for multispecies family protein [25,26]. A related study involving photosynthetic *Bradyrhizobia* (strains BTAi1 and ORS278), reported the absence of nod genes [27]. However, the *nif*U gene is present in all *R. tropici* genomes. Therefore, it appears that some rhizobial genomes, including the strain ‘10ap3’ genome, lack the common *nod* and *nif* genes. It is therefore possible that in some legumes such as pigeonpea, canonical *nod*ABC genes as well as typical lipochito-oligosaccharidic Nod factors (that are encoded by the *nod*ABC genes) which subsequently bind to kinase-like receptors of the host plant, are not necessary for symbiosis. This is partly because the rhizobial strain ‘10ap3’ demonstrated symbiotic efficiency with pigeonpea [20]. Second, some unique rhizobia could employ alternative biochemical pathways to initiate symbiosis using purine derivatives to trigger the formation of nodules [28]. Third, the observed absence of the common nodulation genes is probably because they were not detected in the draft genome using specific primers for the strains of *Rhizobium tropici*.

The genome of the rhizobial strain ‘10ap3’ contained various genes and only one nodulation gene (*nol*R) that was located at contig 12 on the chromosome, similar to *R. tropici* CIAT899, Rhizobium sp. PRF18 and *R. leguminosarum* [25]. The genes that are required for purine biosynthesis that encode inosine-5-monophosphate, AMP and adenylosuccinate were also present on the chromosome of the rhizobial strain ‘10ap3’. Inosine-5-monophosphate and adenylosuccinate induce nodule formation through the purine biosynthesis precursor process [29,30,31]. Purine biosynthesis might be the alternative pathway used by the ‘10ap3’ strain to initiate nodulation on pigeonpea as the common nod genes (*nod*ABC) responsible for nodulation are present on this chromosome [27,32].

The genome size of the rhizobial strain ‘10ap3’ (6.2 Mb) agreed with the findings that were reported previously for *R. tropici* CIAT899 (6.7 Mb) in a similar study [25]. The overall G + C content in each of these genomes was approximately 60.0%, which was comparable with that for the *R. leguminosarum viciae* strain 3841 at 61% [25,32]. One of the possible explanations for this near-identical amount of G + C content is that the strains belong to the same genus (*Rhizobium*) and they are positioned next to each other on both the taxonomic (16S rRNA) and housekeeping (*rec*A) phylogenies or are in the same cluster [33].

For the complete process of nitrogen fixation to occur, successful nodule formation by the rhizobia is necessary as the first step. There is a positive linear correlation between nodulation and the expression of the gene responsible for the purine synthesis. The amount of purine expressed determines the ability of the Rhizobium to form and occupy nodules. Other purine precursors such as inosine and AMP also promote nodulation while adenine promotes strain competitive nodulation [34]. This implies that the gene for the purine biosynthesis pathway (PurL) can control competitive nodulation abilities of rhizobia by monitoring the accumulation of certain purine precursors. These same genes responsible for the purine pathway were also expressed in *Sinorhizobium fredii* during competitive nodulation [34]. The presence of the genes that assist in oxidative stress and tolerance in the strain ‘10ap3’ genome suggests that this strain may tolerate abiotic stresses, such as soil moisture stress, high soil temperature and low soil pH, which are generally associated with the development of most legume cropping systems in South Africa [35,36]. The possible commercialization of this rhizobial strain will benefit legume growers in situations where these abiotic stresses hamper optimum productivity of legumes. In the future, there may be merit in evaluating the symbiotic efficiency of the rhizobial strain ‘10ap3’ under a range of soil moisture stress conditions to determine the threshold at which the strain functions normally.

Apart from possessing the genes that are associated with abiotic stresses, the genome of the strain ‘10ap3’ is equipped with a range of subsystem features that are responsible for various functions. The functions including iron acquisition and metabolism or siderophore aerobactin, which is responsible for iron chelator utilization protein and the ferric aerobactin ABC transporter [37,38,39]. Previous studies showed that iron is critical for the formation of bacterial biofilms since it controls surface activities and can stabilize the polysaccharide matrix [40]. When there is iron deficiency, the hydrophobicity of the microbial surface diminishes significantly leading to alterations in the composition of the surface proteins, hence resulting in restrictions in the formation of biofilms [41]. The siderophores are made up of low (600−1000 Da) molecular weight molecules that possess a high affinity for the ferric ions (Fe^3+^) but low affinity for the ferrous ions (Fe^2+^) that are produced by bacteria under iron deficiency conditions but whose production is suppressed when iron is available. Moreover, siderophores can also complex with several other essential elements such as cobalt, manganese, molybdenum and nickel to avail these metals to rhizobia (or microbes) [42]. In general, iron in the rhizosphere is acquired by plants by acidification through proton extrusion or chelation by secreting complexing molecules (including siderophores, phenolics and carboxylic acids, among others) or the reduction process through secreting compounds that have reducing properties (or reductase activity) [42]. Therefore, this rhizobial strain has potential as a bio-inoculant particularly in soils that are constrained by iron deficiency since it appears to have an ecological advantage for survival in the rhizosphere.

In comparison with the genomes of two other strains, namely, *B. japonicum* (USDA110) and *R. leguminosarum viciae* (strain 3841) that have one rRNA and three rRNAs, respectively, the strain ‘10ap3’ genome also contained three rRNAs [27,32]. However, the rRNA is a non-coding sequence, which is available and useful for cellular function. The RNA contains the genetic material and information that can be translated into proteins by ribosomes [43]. The strain ‘10ap3’ genome also revealed 45 tRNAs, which was comparable to the 52 tRNAs that were reported for *R. leguminosarum viciae* [31].

In conclusion, the successful sequencing of the rhizobial strain ‘10ap3’ genome revealed the absence of the common *nod*ABC nodulation genes suggesting that an alternative pathway involving a purine derivative was involved in the symbiotic association with pigeonpea. This observation was consistent with findings that were reported in other similar studies involving both the *Rhizobium* and *Bradyrhizobium* strains. The genome of the strain ‘10ap3’ also possessed some genes that are associated with abiotic stresses and mineral nutrient acquisition, thus making it a candidate for future formulation of commercial inoculants for pigeonpea. It will be interesting to perform the WGS of the novel rhizobial genera (*Paraburkholderia* and *Phyllobacterium*) that are associated with nitrogen fixation in pigeonpea [6]. From a perspective of legume crop production, a comparison of the genome composition of these distinct genera might shed some light on the variation in symbiotic compatibility and efficiency with various legumes. Such new information might be useful in the formulation of commercial inoculants in future.

## Figures and Tables

**Table 1:** The general features of the rhizobial strain ‘10ap3’.

Genome Feature	Rhizobial Strain ‘10ap3’
Genome size (bp)	6,297,373
G + C content (%)	60
Ribosomal RNA operons	3
Transfer RNAs	45
Protein coding genes/sequences	5961
Assigned functionality (%)	5833
Genes (total) (%)	6013
CDSs (total) (%)	5961
Genes (coding) (%)	5833
CDSs (with protein) (%)	5833
Number of plasmids	0
ncRNAs	4
Pseudogenes	128

bp = base pairs, CDS = coding sequences, G + C = guanine + cytosine, ncRNAs = non-coding ribosomal nucleic acids.

## Data Availability

Additional data are available on request.
