# Correction: Bopape et al. The Genome of a Pigeonpea Compatible Rhizobial Strain ‘10ap3’ Appears to Lack Common Nodulation Genes. *Genes* 2023, *14*, 1084

**DOI:** 10.3390/genes15010074

**Published:** 2024-01-05

**Authors:** Francina L. Bopape, Ahmed Idris Hassen, Rogerio M. Chiulele, Addmore Shonhai, Eastonce T. Gwata

**Affiliations:** 1 Agricultural Research Council, Plant Health and Protection (ARC-PHP), Private Bag X134, Pretoria 0121, South Africa; 2 Department of Plant and Soil Sciences, Faculty of Science, Engineering and Agriculture, University of Venda, Private Bag X5050, Thohoyandou 0950, South Africa; 3 Centre of Excellence in Agri-Food Systems and Nutrition, Eduardo Mondlane University, 5th Floor, Rectory Building, 25th June Square, Maputo 1100, Mozambique; chiulele.rogerio@gmail.com; 4 Faculty of Agronomy and Forestry Engineering, Eduardo Mondlane University, Julius Nyerere Avenue, Maputo 1100, Mozambique; 5 Department of Biochemistry and Microbiology, Faculty of Science, Engineering and Agriculture, University of Venda, Private Bag X5050, Thohoyandou 0950, South Africa

## Addition of an Author

**Prof. Dr. Ahmed Idris Hassen** was not included as an author in the original publication [1]. The corrected Author Contributions statement appears here.

The new authorship and affiliations are as follows:

Francina L. Bopape ^1,2^, Ahmed Idris Hassen ^1,2^, Rogerio M. Chiulele ^3,4^, Addmore Shonhai ^5^ and Eastonce T. Gwata ^2,^*

1. Agricultural Research Council, Plant Health and Protection (ARC-PHP), Private Bag X134, Pretoria 0121, South Africa
2. Department of Plant and Soil Sciences, Faculty of Science, Engineering and Agriculture, University of Venda, Private Bag X5050, Thohoyandou 0950, South Africa
3. Centre of Excellence in Agri-Food Systems and Nutrition, Eduardo Mondlane University, 5th Floor, Rectory Building, 25th June Square, Maputo 1100, Mozambique; chiulele.rogerio@gmail.com
4. Faculty of Agronomy and Forestry Engineering, Eduardo Mondlane University, Julius Nyerere Avenue, Maputo 1100, Mozambique
5. Department of Biochemistry and Microbiology, Faculty of Science, Engineering and Agriculture, University of Venda, Private Bag X5050, Thohoyandou 0950, South Africa

* Correspondence: ectgwata@gmail.com

Ahmed Idris Hassen co-supervised the first author (Francina L. Bopape), contributed in the analysis of whole genome sequence, interpretation of results, and review of manuscript draft.

New Author Contributions: Conceptualization, E.T.G. and F.L.B. Methodology, F.L.B., A.S. and E.T.G. Analysis of data, F.L.B. and A.I.H. Writing—original draft preparation, R.M.C. and E.T.G. Leadership responsibility for the research activity execution, including mentorship external to the core team, critical review and commentary—including pre-publication stages, F.L.B., A.I.H., A.S. and E.T.G. Writing—review and editing, E.T.G. All authors have read and agreed to the published version of the manuscript.

The authors state that the scientific conclusions are unaffected. This correction was approved by the Academic Editor. The original publication has also been updated.
